# The preparedness of urologists to manage cardiopulmonary arrest during robot-assisted surgery

**DOI:** 10.1007/s11701-026-03244-5

**Published:** 2026-02-24

**Authors:** Johnny Wang, Narmina Khanmammadova, Kristene Myklak, Ralph K. Gomez, Tuan Thanh Nguyen, Dat Tien Nguyen, Mohammed Shahait, David I. Lee

**Affiliations:** 1 Department of Urology, University of California, Irvine, Orange, CA USA; 2 Department of Urology, University of Medicine and Pharmacy at Ho Chi Minh City, Ho Chi Minh City, Vietnam; 3 Department of Urological Oncology Surgery, Binh Dan Hospital, Ho Chi Minh City, Vietnam; 4 Golden State Urology, CA Fremont, USA

**Keywords:** Cardiopulmonary arrest, Robot-assisted surgery, Intraoperative emergencies, Minimally invasive surgery

## Abstract

More patients with significant comorbidities and greater perioperative risk are being selected for robot-assisted surgery (RAS) in urology. Cardiopulmonary arrest (CPA) during RAS is an intraoperative complication that poses unique challenges. This exploratory study surveyed practicing urologists to describe preparedness to manage CPA events during RAS. An expert-developed survey was distributed via social media platforms and during international urology conferences between June 2023 and March 2025. The questionnaire assessed demographic characteristics, training background, exposure to CPA during RAS, and institutional preparedness. A total of 50 responses were included in the final analysis. Among 50 respondents, 94% were male, 74% were over 35 years old, and 56% practiced in the United States. Over half (56%) were fellowship-trained. CPA events during RAS were most commonly witnessed by respondents during residency training (76%). However, 78% had never received formal instruction on management of CPA during RAS. Of those who had, training occurred during residency (12%), fellowship (6%), or instructional courses (12%). Most (80%) were unaware of any institutional protocols for CPA during RAS. Respondents selected similar initial management steps and post-arrest strategies across hypothetical scenarios for CPA during pelvic and renal RAS. These findings identify variability in self-reported training exposure and protocol awareness, suggesting areas where structured education and institutional intervention may be considered.

## Introduction

With the growing adoption of robot-assisted surgery (RAS) in urology, a larger proportion of patients with significant comorbidities are now being considered for these procedures. While RAS offers improved outcomes and shorter convalescence time, it also introduces unique intraoperative risks [1, 2]. Concerns include limited physical access to the patient, poor proficiency in emergency undocking, and lack of real-world experience in crisis management in the operating room [3, 4].

Cardiopulmonary arrest (CPA) has long been recognized as a public health concern. In the United States (US), one event occurs every 90 seconds [5]. CPA during the perioperative period, though rare, remains a lethal event, with reported mortality rates exceeding 50% [6]. Data from over 250 hospitals in the United States encompassing 1.3 million surgical cases revealed that approximately 1 in 203 patients required intraoperative cardiopulmonary resuscitation (CPR) [7]. Though incidence is higher in cardiothoracic surgery compared to abdominal surgery (1 in 33 vs. 1 in 258), survival beyond 30 days remains dismal nonetheless [8].

In the operating room, the risk of CPA stems from the intense demands of surgery and anesthesia placed on high-risk patients with already diminished physiologic reserve. Despite the availability of trained personnel and advanced monitoring, CPA during surgery is still underrepresented in CPR protocols, which are often developed for out-of-hospital scenarios [9].

In the event of CPA during RAS, the need for early recognition and initiation of high-quality CPR is essential to survival [10]. The aim of this exploratory, hypothesis-generating study is to describe training exposure, practice patterns, institutional protocols, and decision-making preferences related to management of critical CPA events during RAS.

## Materials and methods

A survey instrument consisting of 22 questions in the English language was developed iteratively by a group of urologists with extensive experience in minimally invasive urologic surgery. The survey was designed to characterize self-reported experiences and preferences rather than to assess technical performance or clinical outcomes. Questions were initially drafted by members of the research team (N.K., T.T.N., D.T.N.), reviewed by the senior authors (M.S., D.I.L.), and refined through group discussion until consensus was achieved. Although the final instrument did not undergo formal validation, all included questions were designed to be concise and directly applicable to RAS workflows in urology.

For the purposes of the survey, CPA was defined as a loss of effective circulation requiring active resuscitation. The survey consisted of questions in key domains including demographics (i.e., age, gender, race, and geographic location), clinical practice (i.e., years in practice, practice setting, case volume), formal training, and institutional protocols. Also included were questions to assess aspects of decision-making for theoretical scenarios of CPA during upper tract and lower tract surgeries, such as off-clamp renal RAS, on-clamp renal RAS, and pelvic RAS.

Following approval from the Institutional Review Board (#2631), the survey was distributed between June 2023 and March 2025 through online social media and in-person during international urology conferences. The REDCap (Vanderbilt University, Nashville, TN) data platform was used to collect and securely store survey responses. Data was analyzed using IBM SPSS Statistics for Windows, Version 29.0 (IBM Corp., Armonk, NY, USA). Results are presented as numbers and percentages. Questions with multiple choice answer formats have percentages that sum to more than 100%.

## Results

### Cohort characteristics

**Fig. 1:**
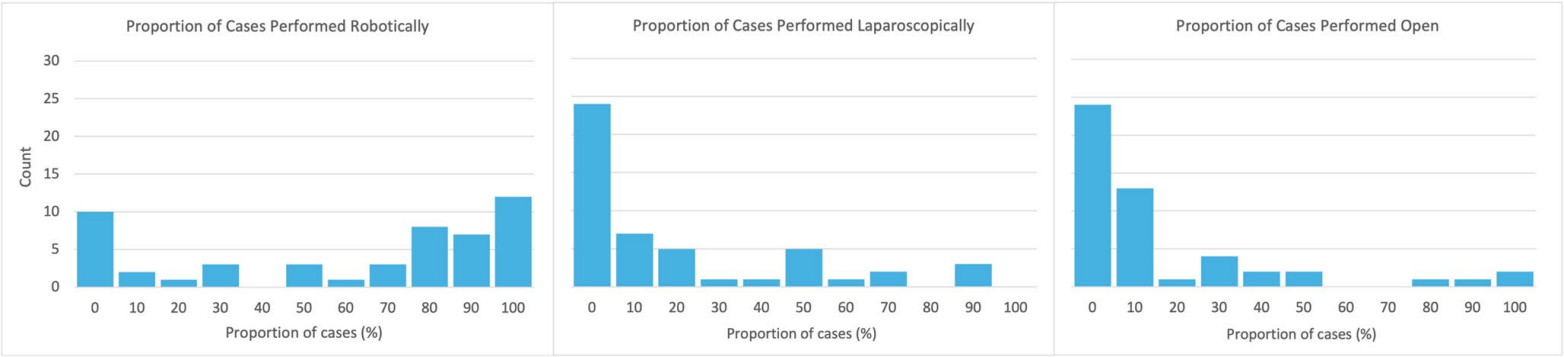
Distribution of responding prostate and kidney cancer surgeons who perform cases robotically, laparoscopically, and open

Fifty practicing urologists completed the survey (Table 1). Most were male (94%), over 35 years old (74%), and residents of the United States (56%). Over half of the respondents completed fellowship training (56%), with subspecialization in robotic/laparoscopic surgery (20%), urologic oncology (18%), or endourology (25%). Practice settings spanned academic or university hospitals (77.6%), non-academic public hospitals (6.1%), and private practice (16.3%). Of the respondents, 43 (86%) reported current access to a daVinci robot. In practice, 34 (68%) reported performing > 3 RAS cases per month, whereas 10 (20%) performed none. RAS was the preferred approach for prostate and kidney cancer surgeries, as 34 (68%) surgeons reported performing ≥ 50% of their prostate and kidney surgeries robotically (Fig. 1). A large proportion of surgeons reported never using open surgery (48%) or never using laparoscopy for these cases (48%).

**Table 1:** Characteristics of surveyed urologists

Variable	Responses, *n* (%)
Male	47 (94%)
**Age (years)**	
≤ 30	9 (18%)
31–35	4 (8%)
36–40	16 (32%)
41–45	9 (18%)
46–50	4 (8%)
> 50	8 (16%)
**Country of residence**	
United States	28 (56%)
Other	22 (44%)
**Years in clinical practice**	
≤ 5	18 (36%)
6–10	6 (12%)
11–15	14 (28%)
16–20	5 (10%)
> 20	7 (14%)
**Fellowship training**	
None	22 (44%)
Endourology	7 (14%)
Urologic Oncology	9 (18%)
Robotic & Laparoscopic Surgery	10 (20%)
Other	2 (4%)
**Practice setting**	
Academic/university hospital	38 (77.6%)
Non-academic public hospital	3 (6.1%)
Private practice	8 (16.3%)

## Training and protocols regarding CPA during RAS

A majority of CPA events during RAS that were witnessed by respondents occurred during residency (76%) or fellowship (16%) training (Table 2). Additionally, most (78%) had no formal training in managing CPA during RAS, with only a small number receiving training through residency (12%), fellowship (6%), or short-term instructional courses (14%). Only 1 in 5 had knowledge of a standardized CPA protocol implemented within their current place of practice.

**Table 2:** Practice patterns and preparedness for cardiopulmonary arrest (CPA) during robotic-assisted surgery (RAS)

Variable	Responses, *n* (%)
**Access to daVinci**^**®**^ **platform**	42 (84%)
**Robotic surgeries performed per month**	
None	10 (20%)
1–2	6 (12%)
3–5	14 (28%)
6–10	7 (14%)
> 10	13 (26%)
**Witnessed CPA events during RAS**	
During residency	38 (76%)
During fellowship	8 (16%)
During practice	4 (8%)
Never	4 (8%)
**Training for management of CPA during RAS**	
During residency	6(12%)
During fellowship	3 (6%)
During short-term training program (≤ 3 months)	0 (0%)
In-person instructional course	4 (8%)
Online instructional course	2 (4%)
On-demand instructional videos	1 (2%)
None	39 (78%)
**Are you aware of institutional protocol for CPA during RAS?**	
Yes	10 (20%)
No	40 (80%)
**Should urologists receive formal training in CPA during RAS?**	
Yes	27 (54%)
No, as it is rare.	4 (8%)
No, as it is covered in Basic and Advanced Life Support courses	19 (38%)
**Should hospital develop protocols to manage CPA during RAS?**	
Yes	39 (78%)
No, as it is rare.	4 (8%)
No, as it is covered in Basic and Advanced Life Support courses	7 (14%)

Roughly half (54%) of respondents believed urologists should receive formal training for management of CPA during RAS (Table 2). Some believed training should not be required due to the rarity of CPA events during RAS (8%) or due to overlap with Basic and Advanced Life Support courses (38%). However, most (78%) supported the notion that hospitals should guide the development and implementation of protocols for management of CPA during RAS.

## Theoretical scenarios

**Fig. 2:**
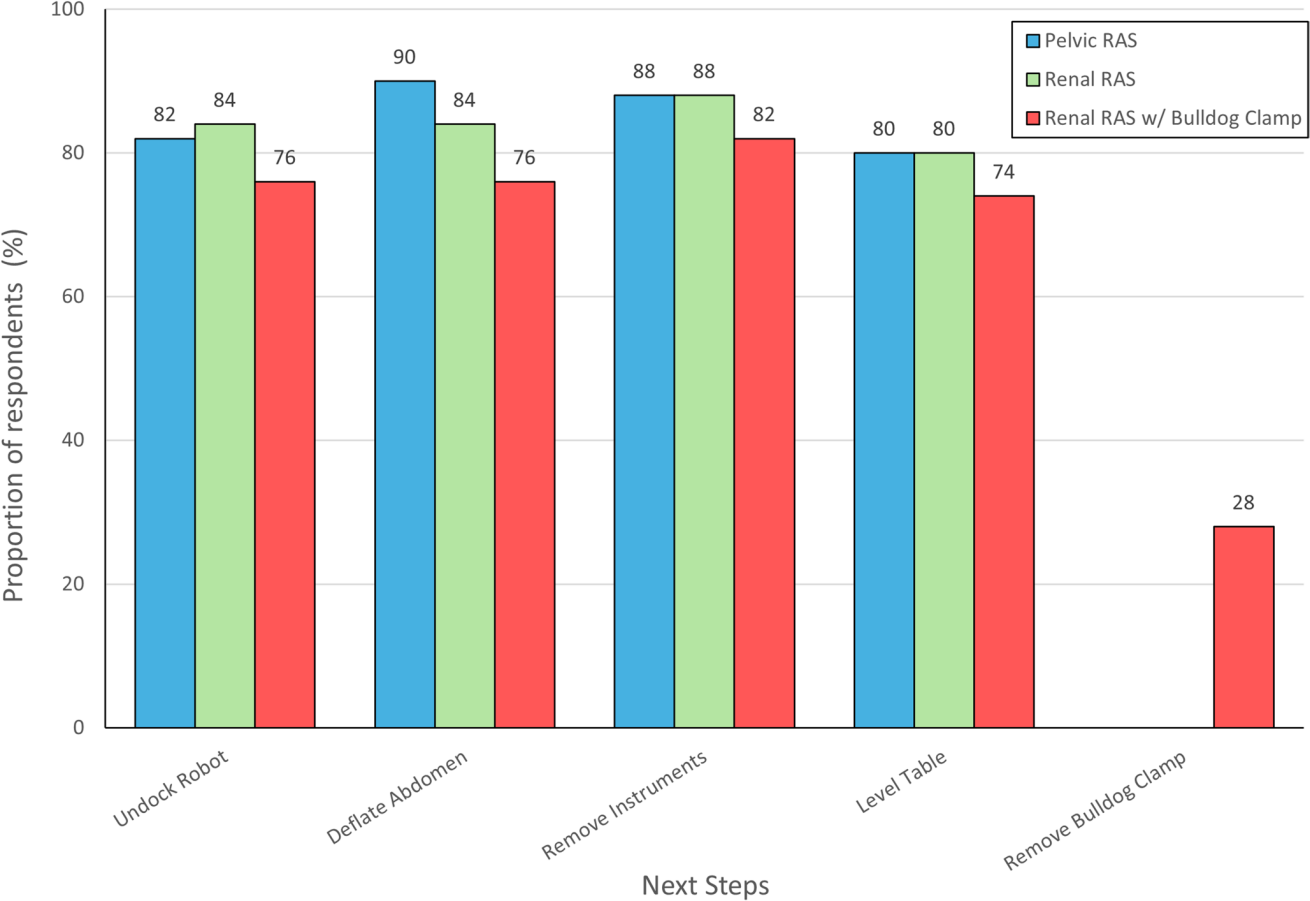
Preferred steps in initial resuscitation of cardiopulmonary arrest (CPA) in robot-assisted surgery (RAS)

During CPA in pelvic RAS, most respondents agreed on the key steps for initial approach to effective CPR: undocking the robot (82%), deflating the abdomen (90%), removing instruments (88%), and leveling the operating Table (80%). This pattern remained true for renal RAS, where 84% supported undocking, 84% supported deflating, 88% supported removing instruments, and 80% supported leveling the table. For CPA during renal RAS with use of a bulldog clamp, 28% supported removing the clamp. Most still endorsed undocking (76%), deflating (76%), removing instruments (82%), and leveling the Table (74%). These results are shown in Fig. 2.

**Fig. 3:**
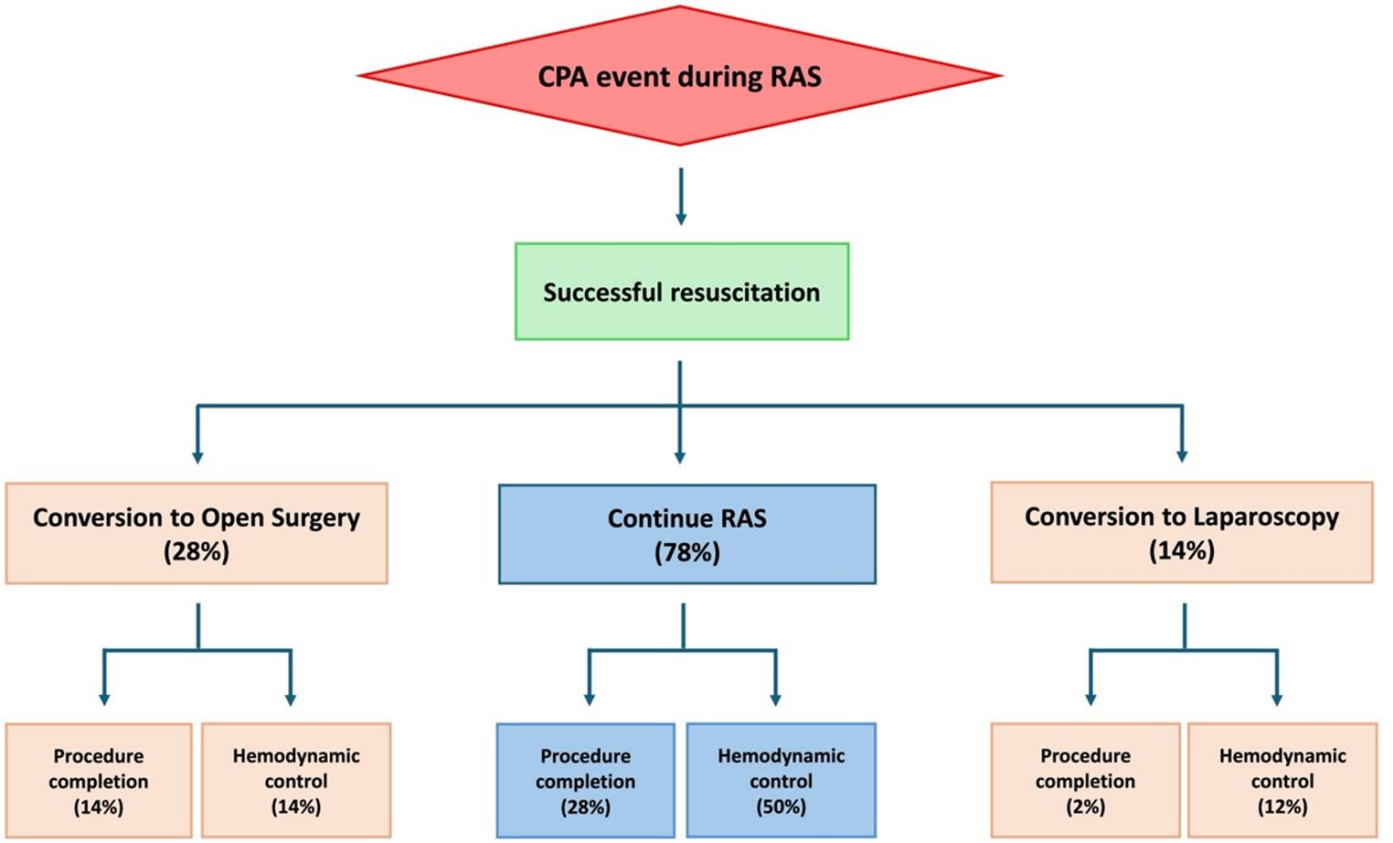
Preferred management strategies after recovery from cardiopulmonary arrest (CPA) during renal robotic-assisted surgery (RAS)

Preferred management strategies after successful recovery from CPA during on-clamp renal RAS varied amongst respondents (Fig. 3). Percentages sum to greater than 100% because participants were allowed to select more than one strategy. Half (50%) of respondents supported continued use of the robot to ensure hemodynamic stability without completion of the surgery, while 28% supported continuation of the planned RAS. On the other hand, fewer surgeons preferred conversion to open surgery for completion of the procedure (14%) or for hemodynamic stabilization without completion of the surgery (14%). A smaller minority (12%) preferred converting to laparoscopy for hemodynamic control.

## Discussion

This exploratory study describes the preparedness of urologists to manage CPA during RAS. Despite widespread integration of RAS within urologic surgery, most respondents reported never receiving formal training in intraoperative CPA management. Additionally, 80% were unaware of any institutional CPA protocols, and 78% endorsed the need for hospital to develop standardized protocols. Together, these findings suggest potential gaps in training and institutional resources relevant to patient safety during RAS.

Though rare, a true CPA event during RAS can have devastating consequences. The management of CPA during RAS is complicated by limited patient access and separation of the surgeon from the operative field. Though most healthcare professionals are trained in Basic or Advanced Life Support, such interventions remain highly time sensitive [11, 12]. Preparing for resuscitation, which requires steps such as undocking the robot, deflating the pneumoperitoneum, removing instruments, repositioning the operating table, and scrubbing in, can introduce critical delays.

An effective response demands rapid, coordinated action from the surgical team. Communication lapses, especially among providers with differing training backgrounds, can hinder timely intervention [13, 14]. Huser et al. demonstrated that multidisciplinary teams (urologists, anesthesiologists, and nursing staff) were able to significantly reduce time to robot undocking and time to initiation of chest compressions through simulation training and structured debriefing [15]. Similar findings have been reported in robotic thoracic and gynecologic surgery [4, 16]. While these studies do not evaluate clinical outcomes, they suggest that structured, team-based training improves coordination during intraoperative emergencies.

Along with team-based training, institutional protocols may provide additional structure during CPA events. Checklists and role-specific algorithms have been proposed as both educational tools and real-time decision aids [17, 18]. In thoracic surgery, the Society of Thoracic Surgeons recommends using adaptable algorithms or checklists tailored to institutional resources and personnel [19]. Professional organizations such as the Society of Urologic Robotic Surgeons (SURS) or North American Robotic Urologic Society (NARUS) are well-positioned to lead similar initiatives within urology.

The variability in responses observed in scenario-based questions may reflect several factors. For example, surgeon experience and case volume differ widely; higher volume robotic surgeons may feel comfortable proceeding robotically after successful resuscitation, while others may prefer conversion for safety [20]. Management decisions are also influenced by the underlying etiology. Conversion to laproscopy or laparotomy should be considered in case of vascular complications, such as hemorrhage, gas embolism, or large vessel injury. However, events less severe than true CPA, such as transient hypotension or arrhythmia, are often successfully managed by anesthesiology without interruption of the procedure. Patient factors, particularly physiological reserve, also impact decision-making. Patients with cardiopulmonary comorbidities like congestive heart failure or chronic obstructive pulmonary disease may not tolerate prolonged pneumoperitoneum or Trendelenburg positioning, especially in the aftermath of an acute insult [21].

The dearth of data regarding CPA during RAS presents a two-fold dilemma. For patients, it may foster misinformation and reduce confidence in RAS [22]. For surgical teams, it hinders consensus on strategies for prevention and intervention. Isolated reports of rare events, such as air embolism during single port transvesical robotic prostatectomy, illustrate how limited case numbers preclude comprehensive root cause analysis [23, 24]. Knowledge gaps may contribute to a false sense of security, as operating room staff may be unclear about their roles during emergency conversion despite high perceptions of preparedness [25]. Under-reporting of CPA events may also occur due to medicolegal concerns, inconsistent definitions, and absence of adverse event registries. Overall, the need for standardized reporting to promote transparency and learning from these events is evident.

This study has several limitations. The modest sample size and convenience sampling approach introduce the potential for selection and recall bias. The prolonged recruitment period may also introduce temporal heterogeneity due to shifting guidelines and practice patterns, though a thorough literature search revealed no significant changes reported in this time period. Moreover, most respondents were male and affiliated with academic centers, which limits generalizability of these findings to the broader urologic community. Additionally, the survey instrument was not formally validated, and responses to hypothetical scenarios have no guarantee of reflecting real-world behavior. Despite these limitations, this study provides an initial description of urologists’ experiences and perspectives regarding CPA during RAS and identifies areas for future investigation and educational development.

## Conclusions

While intraoperative CPA during RAS remains rare, the consequences are often catastrophic. As robotic surgery becomes central to urologic surgery, it is imperative to incorporate emergency preparedness into surgeon training and institutional protocols. Our findings suggest that further development of education initiatives and management protocols may be beneficial.

## Data Availability

Data used in this study is not publicly available due to the inclusion of potentially identifiable participant information. De-identified data may be made available from the corresponding author upon reasonable request.
